# A *C. elegans* MAP kinase pathway is required for wild-type display of an L1-specific surface antigen (*srf-6* is *nsy-1* III)

**DOI:** 10.17912/micropub.biology.000129

**Published:** 2019-07-04

**Authors:** Stephen J. Foley, Zheyang Wu, Samuel M. Politz

**Affiliations:** 1 Department of Chemistry and Biochemistry, Worcester Polytechnic Institute, Worcester, MA; 2 Department of Mathematical Sciences, Worcester Polytechnic Institute, Worcester, MA; 3 Department of Biology and Biotechnology, Worcester Polytechnic Institute, Worcester, MA

**Figure 1.**
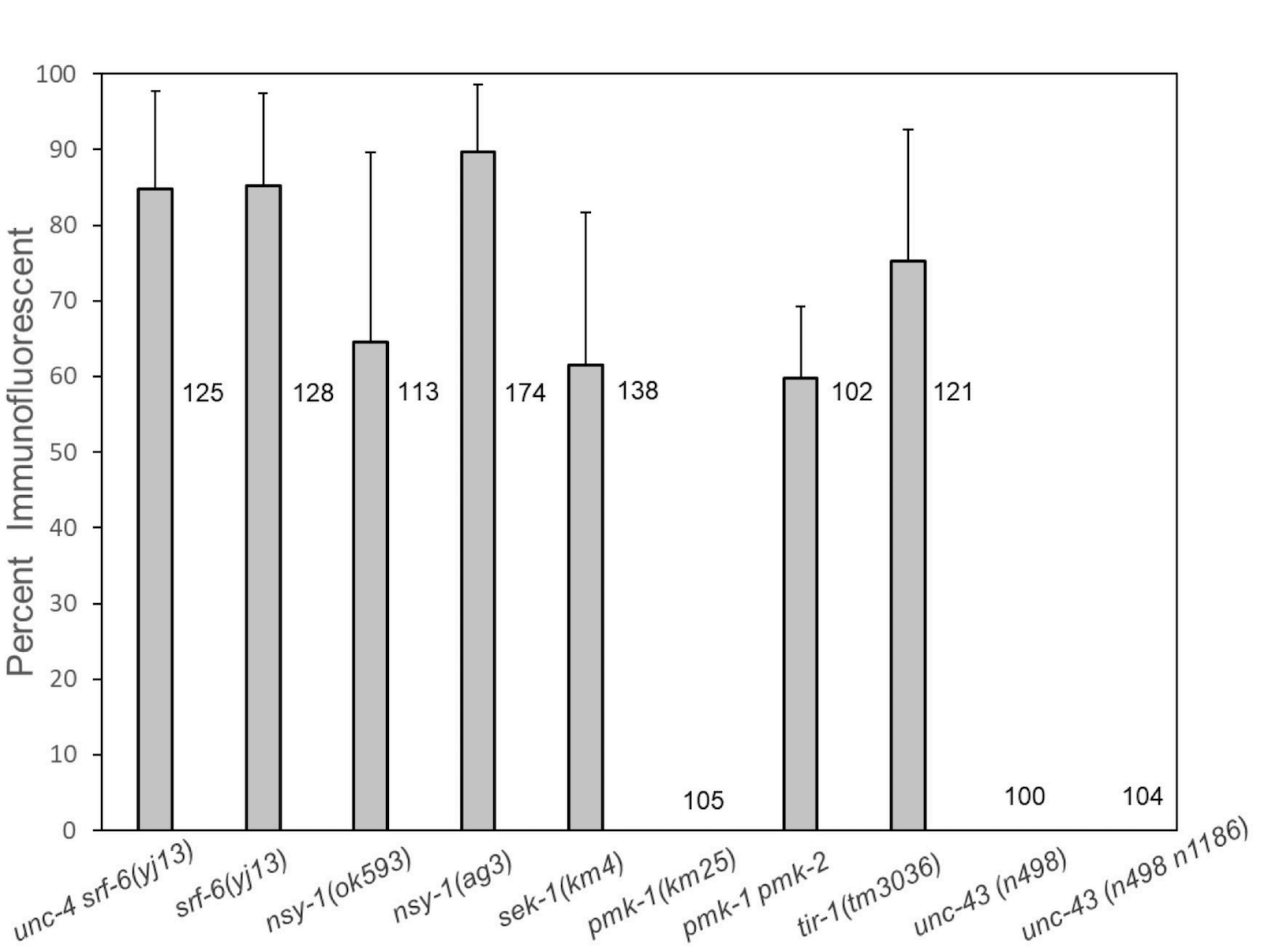
Immunofluorescent staining of *C. elegans* L2-L4 hermaphrodites with monoclonal antibody (mAb) M37. Worms were stained with mAb M37 and FITC-conjugated goat anti-mouse IgM as described (Hemmer et al., 1991; Grenache 1996) and examined in a fluorescent microscope. Histogram bars indicate the percentage of worms showing surface immunofluorescence. Each bar includes results from four biological replicates, except for *unc-43(n498)*, which includes results from three biological replicates. Numbers to the right of each bar indicate total numbers of worms stained. Error bars indicate +/- one standard deviation. In samples of *pmk-1(km25)*, *unc-43(n498)*, and *unc-43(n498 n1186)* L2-L4 stages, no worms showed surface immunofluorescence*.*

## Description

In previous work, we showed that three different *srf-6* mutants carry mutations in gene *nsy-1* (Van Sciver et al., 2019), and that a *srf-6* mutant shows the 2AWC^ON ^phenotype characteristic of *nsy-1* mutants. We also showed that *srf-6(yj13)* fails to complement *nsy-1(ok593)* (Honzel et al., 2019). We concluded that *srf-6* and *nsy-1* are the same gene.

*C. elegans* gene *nsy-1* encodes a MAP kinase kinase kinase (MAPKKK) that carries out the first step in a *C. elegans* MAP kinase pathway (Kim et al., 2002, Sagasti et al., 2001, Tanaka-Hino 2002). The pathway culminates in activation of a MAP kinase that is homologous to the human p38 MAP kinase (Kim et al., 2002). This pathway is involved in at least three biological processes in *C. elegans*, i.e., immune responses to infection by pathogenic microbes (Kim et al., 2002), upregulation of serotonin biosynthesis in the ADF chemosensory neurons in response to pathogen exposure (Shivers et al., 2009, Zhang, Lu, and Bargmann 2005), and determination of asymmetric cell fate in the AWC chemosensory neuron pair (Sagasti et al., 2001).

*C. elegans srf-6* mutants were originally identified by antibody staining at stages L2-L4 with mAb M37, which stains only the L1 stage in wild type *C. elegans* under normal growth conditions (Hemmer et al., 1991, Grenache et al., 1996)*.* To determine whether loss of function in the *C. elegans* p38 pathway also results in staining at stages L2-L4, we tested *nsy-1* (MAPKKK), *sek-1* (MAPKK) and *pmk-1* (MAPK) mutants for mAb M37 staining at these later larval stages. Results are shown in Figure 1.

In these experiments, *nsy-1(ok593)* and *nsy-1(ag3)* also showed staining of L2-L4 stages (Figure 1)*.* Staining of *sek-1(km4)* was also positive. The Toll-interleukin-receptor-like protein TIR-1 is required upstream of *nsy-1* for both immune responses and determination of AWC fate (Couillault et al., 2004, Chuang and Bargmann 2005). Positive antibody staining was also observed in *tir-1(tm3036)* mutants.

It was surprising that no L2-L4 larvae of a mutant strain carrying *pmk-1(km25)* stained (out of a total of 105 worms in 4 biological replicates), because PMK-1 is required as the MAP kinase for immune responses (Kim et al., 2002). However, a requirement for PMK-1 has not been demonstrated for determination of AWC cell fate (Pagano, Kingston, and Kim 2015). It has been shown that function of the TIR-1 NSY-1 SEK-1 pathway is required for determination of AWC fate (Chuang and Bargmann 2005), and that PMK-1 and a second MAP kinase, PMK-2, function redundantly for determination of AWC cell fate (Pagano, Kingston, and Kim 2015). We therefore tested a *pmk-2 pmk-1* double mutant for M37 antibody staining. In contrast to *pmk-1*, the *pmk-1 pmk-2* double mutant showed staining at a level similar to that of *sek-1(km4).*

The *unc-43*-encoded calcium dependent kinase CaMKII is required for upstream activation of the TIR-1 NSY-1 SEK-1 cascade in the AWC neuron, and the level of *unc-43* activity in AWC can determine whether the cell develops the AWC^ON^or the AWC^OFF ^fate (Troemel, Sagasti, and Bargmann 1999). We tested the mutant *unc-43(n498gf)*, which shows a 2AWC^OFF^phenotype, and the 2AWC^ON^mutant *unc-43(n498n1186lf)* for M37 antibody staining (Troemel, Sagasti and Bargmann 1999). Neither of these mutants stained with mAb M37.

## Reagents

*C. elegans* Strains

AT18 *srf-6(yj13)* II

VC390 *nsy-1(ok593)* II

AU3 *nsy-1(ag3)* II

KU4 *sek-1(km4)* IV

KU25 *pmk-1(km25)* II

ZD1006 *pmk-2(qd279qd171) pmk-1(km25)* II

IG685 *tir-1 (tm3036)* III

MT1092 *unc-43(n498)* IV

MT2605 *unc-43(n498) unc-43(n1186)* IV

All strains are available from the *Caenorhabditis* Genetics Center, except for AT18, which will be sent to the CGC.
